# Green synthesis and characterization of selenium nanoparticles: preliminary compatibility assessment with beneficial rhizobacteria and their role in enhancing aggregatum onion (*Allium cepa* L. var. *aggregatum*) seed germination under salinity stress

**DOI:** 10.3389/fpls.2026.1911881

**Published:** 2026-08-21

**Authors:** Gomathi Arivalagan, Utpal Das

**Affiliations:** Department of Horticulture and Food Science, VIT School of Agricultural Innovations and Advanced Learning, Vellore Institute of Technology, Vellore, Tamil Nadu, India

**Keywords:** selenium nanoparticles, biosynthesis, characterization, aggregatum onion, seed germination, salinity stress

## Abstract

The era of nanotechnology has presented a new window of opportunity for sustainable agriculture, and the contribution of nanoparticles (NPs) to stress alleviation of abiotic stresses, with their exceptional physicochemical properties in the biological domain, has been the actual focus of researchers. Selenium nanoparticles (SeNPs) provide a promising nano-based solution to enhancing the delivery of selenium and the early development of plants in agriculture. In this work, the selenium nanoparticles were synthesized using the green synthesis technique with plant leaf extracts of guava and sapota, and thoroughly characterized to verify the physicochemical characteristics. The presence of selenium nanoparticles was confirmed by UV-Visible spectroscopy, and the role of plant-derived phytochemicals in the reduction and stabilization of nanoparticles was identified by FTIR. The amorphous form of the synthesized SeNPs was determined by X-ray diffraction analysis and scanning electron microscopy revealed that the nanoparticles were mostly spherical. Elemental analysis using EDAX confirmed the presence of selenium and thermogravimetric analysis revealed that the nanoparticles were thermally stable. One of the most important abiotic factors which severely limits development and production of aggregatum onion is salinity. However, the responses of plants to selenium nanoparticles under salt stress during seed germination have not been studied in detail. This study focuses on the effect of nanopriming on germination processes by using selenium nanoparticles (SeNPs) in different concentrations on salinity stress during the early seedling stage of germination of aggregatum onion. Nanopriming enhanced the final germination percentage, germination rate, speed of germination, seedling growth, seedling vigor, biomass accumulation, and antioxidant enzyme activity of aggregatum onion under salt stress. The highest germination percentage (93.3%) and superior seedling vigor were recorded at 50 ppm SeNP treatment. The results demonstrated that the use of SeNPs could be a new strategy and helpful approach to improve salinity tolerance in small onion crops.

## 1 Introduction

Nanotechnology provides an alternative way to overcome the limitations of the existing biotic stress management strategies and revolutionize agricultural research. According to Acharya et al. (2019), the application of nanoparticles (NPs) in agriculture significantly improves plant growth and seed germination in various plant species while limiting the use of excessive chemicals. Fertilizers, pesticides, and biostimulants developed using nanotechnology have been reported to increase the efficiency of nutrient uptake, tolerance to stress, and the productivity of crops with low ecological footprints (Chhipa, 2019; Du et al., 2024). The key limitation to crop productivity at the global scale is abiotic stresses like drought, salinity, heat, and heavy metal toxicity. Over 20% of cultivated land and 33% of irrigated agricultural land worldwide are damaged by salinity; as a result, 1.5 million hectares are left uncultivated, costing the agricultural sector over $27.3 billion annually and extending issues with food security (Mehmood et al., 2021). By 2050, 50% of soils are expected to be affected by salt (Shrivastava and Kumar, 2015; Majeed and Muhammad, 2019).

Selenium (Se) is a trace element, but has functional impacts on plant development, metabolism and nutritional value. It is not universally considered an essential element for higher plants, but is becoming a valued element through its capacity to improve physiological performance and adaptability to biotic and abiotic stresses (Arivalagan and Das, 2026). The thin border between positive and adverse levels of traditional selenium fertilizers has consequently prompted the creation of less toxic and more effective selenium delivery systems to support sustainable agriculture (Skalickova et al., 2017; El-Ramady et al., 2020). As a result, enhancing the efficiency and reducing the toxicity of selenium has become one of the main research goals of modern crop nutrition and biofortification initiatives (White, 2016; El-Ramady et al., 2022). Recent breakthroughs in nanotechnology have provided new ways to overcome these difficulties by creating selenium nanoparticles (SeNPs). Compared to bulk or ionic selenium species, selenium in nanoform displays greater bioavailability, controlled reactivity, and lower toxicity. SeNPs, because of their small size (usually 1–100 nm), have distinctive physicochemical characteristics, such as high surface area, enhanced interaction with biological membranes, and the stability that together lead to the excellent biological activity in plant systems (Usman et al., 2020; Zeeshan et al., 2024). The use of green or biogenic synthesis of selenium nanoparticles using plant extracts has received much attention among other synthesis methods. The method does not require the use of toxic chemicals, rendering the process ecologically harmless, economical, and scalable. Moreover, phytochemical capping is beneficial in improving the stability of nanoparticles and biological compatibility, particularly in agriculture (Ramamurthy et al., 2019; Singh et al., 2023). In plants, selenium nanoparticles influence multiple physiological and biochemical processes. When applied using seed priming, soil amendment, or foliar spraying, SeNPs are taken up and slowly converted to bioactive selenium species in plant tissues (Wang et al., 2020). SeNPs enhance the process of chlorophyll biosynthesis, photosynthetic efficiency, and gas exchange parameters at optimal concentrations (Pattnaik et al., 2025). They also promote the action of important antioxidant enzymes like superoxide dismutase, catalase, and peroxidase, thus lowering the accumulation of reactive oxygen species during stressful situations. The overall effect of these processes is enhanced plant vigor, biomass accumulation, and yield stability (Hernández-Hernández et al., 2019; Zeeshan et al., 2024). The antimicrobial activity of SeNPs also helps to protect plants by suppressing phytopathogenic bacteria and fungi by oxidative damage to cellular components and thiol oxidation processes (Ramos and Webster, 2012; Rastogi, 2019). This dual role as a nutrient and a protective agent also contributes to improving the agronomic value of SeNPs. A number of techniques have been developed recently for the application of NPs to improve plant growth and productivity, including seed priming. Priming is the most promising invigoration technique to improve the germination rate and the development of plants, based on the control of hydration and drying to reach rapid germination by enhancing sufficient germinative metabolic processes (Waqas et al., 2019).

Recent reviews have highlighted the broad potential of nanoparticle-based approaches to improve plant growth, productivity, and tolerance to abiotic stresses across economically important crop species while reducing dependence on conventional agrochemicals (Roy et al., 2025a). Among the different nanomaterials investigated, plant-mediated selenium nanoparticles have emerged as a promising strategy, with recent studies demonstrating their ability to enhance salinity tolerance in several crop species (Sharif et al., 2026); however, most reports employ different botanical extracts and crop systems. The use of *Psidium guajava* leaves as a reducing and stabilizing agent for SeNP synthesis has not been explored for seed-priming applications, and no information is available on the response of *Allium cepa* var. *aggregatum* to guava-derived SeNPs under salt stress. Furthermore, it is unclear how nanopriming affects physiological and biochemical changes that occur during the germination stage. Therefore, the present study was undertaken to develop a guava-mediated green synthesis method for SeNPs and to evaluate its efficacy in improving germination and early seedling performance of aggregatum onion under salinity conditions. The results will add important knowledge of the role of biogenic SeNPs which facilitates germination efficiency and early growth without causing possible phytotoxic effects which will contribute to the wider use of nanotechnology-based solutions in sustainable agriculture, stress management, and crop productivity enhancement.

## 2 Materials and methods

### 2.1 Chemicals

The fresh leaves of guava and sapota were collected from Sevoor Farm, VIT, Vellore, Tamil Nadu. All the other chemicals used were of analytical grade.

### 2.2 Synthesis and purification of SeNps

**Figure 1:**
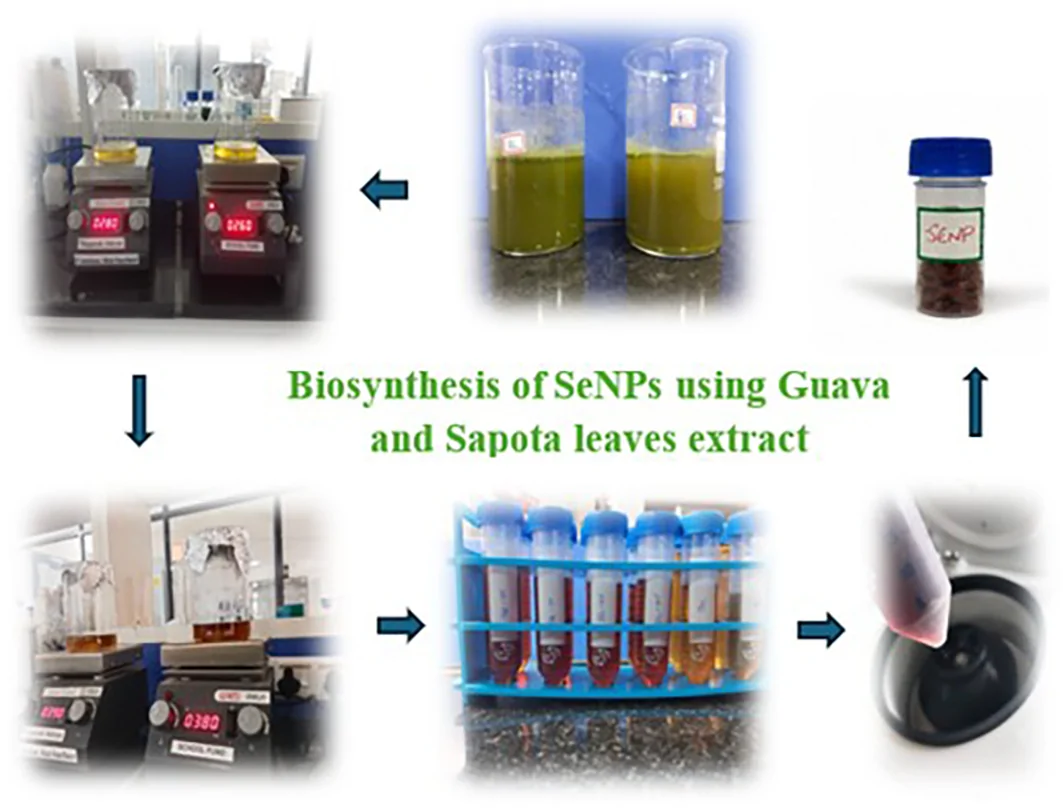
Biosynthesis of selenium nanoparticles using guava and sapota leaves extract.

The leaves were washed thrice using distilled water, shade-dried for 3 days and ground into a fine powder. Two grams of each leaf powder were mixed with 200 mL of distilled water and blended for 30 minutes and filtered for the subsequent process. One hundred milliliters (100 mL) of leaf extract was mixed with 90 mL of 25 mM sodium selenite solution; the mixture was placed on an orbital shaker in the dark for 3 hours at 210 rpm. The mixture was centrifuged at 12,000 rpm for 20 mins, and the pellets were washed three times and then air-dried to obtain pure SeNPs (**Figure 1**).

### 2.3 Characterization of SeNPs

Selenium nanoparticles (SeNPs) were synthesized using aqueous leaf extracts of guava (*Psidium guajava*) and sapota (*Manilkara zapota*). The synthesized SeNPs were characterized using UV–visible spectroscopy (Genesys 180, Thermo Scientific, USA) over a wavelength range of 200–800 nm to confirm nanoparticle formation. The morphology and elemental composition of the nanoparticles were analyzed using Scanning Electron Microscopy (SEM; FEI Quanta, FEI, Netherlands) coupled with Energy-Dispersive X-ray Spectroscopy (EDX). The crystalline structure of the synthesized SeNPs was determined by X-ray Diffraction (XRD). Fourier Transform Infrared Spectroscopy (FT-IR) was employed to identify the functional groups in the leaf extracts responsible for the reduction and stabilization of SeNPs. The hydrodynamic particle size and size distribution were determined using Dynamic Light Scattering (DLS), while the surface charge and colloidal stability were assessed by zeta potential analysis using a nanoparticle analyzer (Particle Metrix ZetaView PMX-130, Mono Laser). Based on the comparative characterization results, guava-mediated SeNPs exhibited superior physicochemical characteristics and were therefore selected for subsequent germination experiments.

### 2.4 Germination test

A preliminary optimization study was conducted to determine the appropriate concentrations of SeNPs and NaCl for the germination experiment. Based on these preliminary results, SeNP concentrations of 25 and 50 ppm and a salinity level of 25 mM NaCl were selected for further investigation. The germination experiment was carried out to evaluate the effect of guava-mediated SeNP seed priming on germination and early seedling growth of aggregatum onion under salinity stress using the Paper Towel Method recommended by the International Seed Testing Association (ISTA) (Kumar Kurdekar et al., 2024). The objective of this experiment was to assess the efficacy of the optimized green-synthesized SeNP formulation in mitigating salt stress during germination rather than to compare different selenium sources or plant extracts.

Seeds of aggregatum onion (*Allium cepa* var. *aggregatum*) cv. CO (On) 5 were obtained from the Tamil Nadu Agricultural University, Coimbatore. Seeds were disinfected with 70% ethanol followed by surface sterilization using 1% (v/v) sodium hypochlorite for 2 min and subsequently rinsed thoroughly with deionized water (Gamit et al., 2025). A seed-to-solution ratio of 1:2 (w/v) was maintained during priming. Seeds were primed with guava-mediated SeNP suspensions at 25 ppm and 50 ppm, while hydropriming with distilled water served as the control treatment. Priming was performed by soaking the seeds for 6 h on an orbital shaker at 120 rpm under ambient laboratory conditions. Following priming, the seeds were shade-dried at room temperature until they regained their original moisture content.

The germination experiment was conducted with 15 seeds per replicate and three independent biological replicates, resulting in 45 seeds per treatment. The seeds were placed between two moistened corrugated brown paper towels. Distilled water was used for the non-saline treatment, whereas 25 mM NaCl solution was used to impose salinity stress. The paper towels were rolled into uniform cylinders, wrapped with butter paper, secured with rubber bands, and incubated in a seed germinator maintained at 25 ± 2 °C and 75 ± 5% relative humidity. The experiment consisted of seven treatments: (T1) SeNP priming @ 25 ppm under normal conditions, (T2) SeNP priming @ 50 ppm under normal conditions, (T3) SeNP priming @ 25 ppm + 25 mM NaCl under salinity stress, (T4) SeNP priming @ 50 ppm + 25 mM NaCl under salinity stress, (T5) untreated control under normal conditions, (T6) hydropriming under normal conditions, and (T7) 25 mM NaCl under salinity stress. Germination was monitored daily until the final count of germinated seed was reached, based on visible radicle emergence. The data were collected from 3^rd^, 7^th^, 10^th^ and 14^th^ days after sowing of seeds. The final germination percentage and seedling growth parameters were assessed to evaluate the efficacy of SeNP priming in enhancing germination and early seedling establishment under salinity stress.

#### 2.4.1 Optimization of nanoparticle concentrations for seed priming

To attain a stock solution of 1000 mg L^-1^, the nanoparticles were dispersed in double-distilled water and then serially diluted into working solutions of 25 and 50 ppm. To achieve the uniform distribution of the nanoparticles, the solutions were subjected to probe ultrasonication at 40 kHz for a duration of 20 minutes (Kumar et al., 2025).

#### 2.4.2 Germination %

The percentage of germination was calculated based on the total count of emerged seedlings using standard procedures.


$$\text{Germination }\%=\frac{\text{Total no},\text{ of normal seedlings}}{\text{Total no}.\text{ of seeds}}\times 100$$

#### 2.4.3 Growth traits

On the 14th day of the trial, 10 healthy seedlings were arbitrarily selected from each replication to calculate seedling length. The primary root was measured from the collar area to its tip, and the shoot length was measured from the collar to the apex of the shoot. The overall seedling length was measured and the average values were reported in centimeters (cm). The selected seedlings underwent shade-drying and subsequent oven drying at 50°C for 2 hours. Dry biomass of ten seedlings from each group was measured and expressed in milligrams.

#### 2.4.4 Estimation of seedling vigor index I and II

Seedling vigor indices were determined following the method described by (Abdul-Baki and Anderson, 1973).


Vigour index I=Germination (%)×Total seedling length (cm)

Vigour index II=Germination (%)×Dry matter production (mg)

#### 2.4.5 Speed of germination

SOG was calculated in accordance with (Maguire, 1962).


$$\text{Speed of germination}=\;\frac{\text{X}1}{\text{Y}1}+\;\frac{\text{X}2-\text{X}1}{\text{Y}2}+\ldots \ldots \ldots \ldots \ldots .+\;\frac{\text{Xn}-\text{Xn}-1}{\text{Yn}}$$

Where, X_1_ – count of seeds germinated at first assessment.

X_2_ – count of seeds germinated at the first assessment.

X_n_ - count of seeds germinated at the n^th^ assessment.

Y_1_ - number of days from sowing to the first count.

Y_2_ - number of days from sowing to the second count.

Yn - number of days from sowing to nth count.

**Figure 2:**
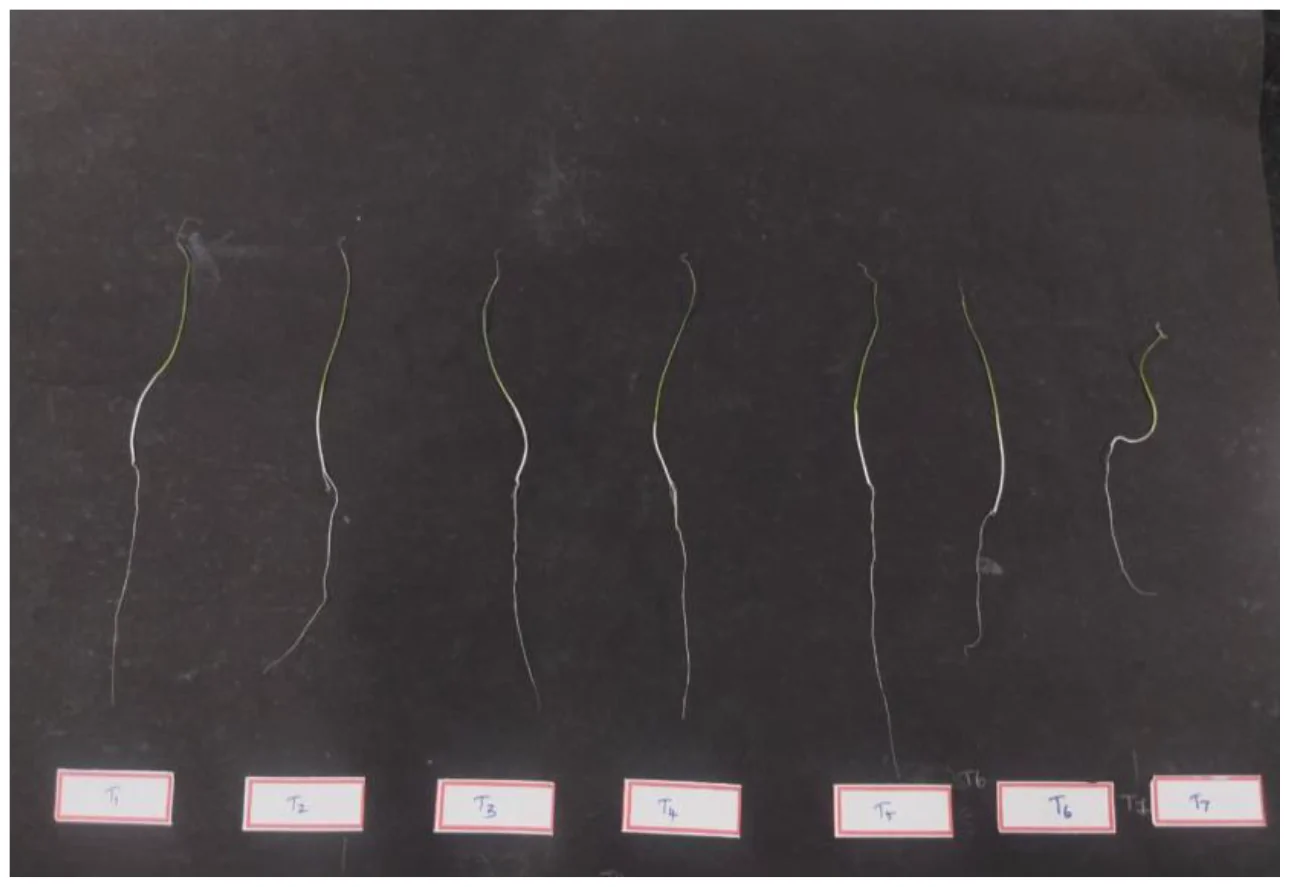
The effect SeNPs seed priming on early seedling growth of aggregatum onion under salinity stress.

To determine the most effective SeNPs on the Aggregatum Onion seeds germination under salinity stress (25Mm NaCl) (**Figure 2**). Among the SeNPs synthesized by green methods, derived SeNPs were used at 2 different concentrations (25ppm and 50ppm). The study was designed to investigate the effects of Selenium nanoparticles on Aggregatum onion seedling growth, comprising 7 treatments, including the control, Selenium nanoparticles primed seeds in three replicates laid in a Completely Randomized Design (CRD) (**Table 1**).

**Table 1:** Treatment details.

S. no.	Treatments
T_1_	SeNPs @25ppm
T_2_	SeNPs @50ppm
T_3_	SeNPs @25ppm + 25mM NaCl
T_4_	SeNPs @50ppm + 25mM NaCl
T_5_	Control
T_6_	Hydropriming
T_7_	25mM NaCl

### 2.5 Biochemical analysis

Freshly grown 14-day-old seedlings were harvested and were immediately subjected to biochemical analysis. To extract enzymes, 0.5 g of fresh tissue was homogenized in ice-cold 50 mM phosphate buffer (pH 7.8) and centrifuged for 20 min at 4 °C at 12,000 × g. The supernatant was used for the determination of antioxidant enzyme activities.

#### 2.5.1 Superoxide dismutase activity

The SOD activity was determined as described by Giannopolitis and Ries (1977) with a slight modification. The reaction mixture contained 50 mM phosphate buffer (pH 7.8), methionine (13 mM), nitro blue tetrazolium (NBT) (75 μM), EDTA (0.1 mM), riboflavin (2 μM), and enzyme extract. The reaction was started by illuminating the tubes with fluorescent light for 10 min and stopped by turning off the light source. Absorbance was measured at 560 nm. The units of SOD activity were defined as the amount of enzyme that caused 50% inhibition of the photoreduction of NBT and expressed in U mg^-^¹ protein.

#### 2.5.2 Peroxidase activity

The POD activity was measured by the Change and Maehly (1955) method, slightly modified. The reaction mixture contained 50 mM of phosphate buffer (pH 6.8), 20 mM guaiacol and 40 mM H_2_O_2_, along with enzyme extract. The reaction was started with the addition of H_2_O_2_ and the absorbance at 470 nm for the formation of tetraguaiacol was measured for 3 min on a UV–Visible spectrophotometer. The activity of peroxidase was determined by the rate of increase in absorbance and expressed as U mg^-^¹ protein, with one unit being defined as a change in absorbance of 0.01 units per minute under the assay conditions.

#### 2.5.3 Catalase activity

The activity of CAT was estimated according to Aebi (1984). Enzyme extract was added to the reaction mixture which comprised 50 mM phosphate buffer (pH 7.0) and 15 mM H_2_O_2_. The absorbance was measured at 240 nm for 1 min for the decomposition of H_2_O_2_ with a UV–Visible spectrophotometer. The activity was reported as μmol H_2_O_2_ decomposed min−1 mg−1 protein.

#### 2.5.4 Lipid peroxidation (MDA)

The malondialdehyde (MDA) content was estimated as per the method of Heath and Packer (1968). Fresh tissue (0.5 g) was homogenized in 0.1% trichloroacetic acid (TCA) and centrifuged. 4 mL of TCA with 0.5% TBA solution was added to 1 mL of supernatant and heated for 30 min at 95 °C. The reaction was stopped in an ice bath and centrifuged. The absorbance was measured at 532 and 600 nm. MDA concentration was calculated using an extinction coefficient of 155 mM^-^¹ cm^-^¹ and expressed as μmol g^-^¹ fresh weight.

#### 2.5.5 Hydrogen peroxide

Hydrogen peroxide concentration was determined following the method of Velikova et al. (2000). The fresh tissue was homogenized in 0.1% TCA and centrifuged. The reaction mixture consisting of extract, phosphate buffer (pH 7.0) and potassium iodide (KI) was incubated in the dark and absorbance was measured at 390 nm. The concentration of H_2_O_2_ was determined by a standard curve and expressed as μmol g-1 fresh weight.

#### 2.5.6 Proline content

Free proline was measured by the method of Bates et al. (1973). Fresh tissue (0.5 g) was extracted with 3% sulfosalicylic acid. The extract was mixed with acid ninhydrin and glacial acetic acid and heated at 100 °C for 1 h. Upon cooling, toluene was added and the upper phase containing the chromophore was harvested. The absorbance was determined at 520 nm. The concentration of proline was determined by a standard curve and expressed in mg g^-^¹ fresh weight.

#### 2.5.7 Total phenolic content

The total phenolic content was estimated according to the procedure of Folin–Ciocalteu method of Singleton and Rossi (1965). The extract was added to Folin–Ciocalteu reagent, sodium carbonate solution and incubated at room temperature. The absorbance was measured at 765 nm. Gallic acid was used as the standard and results were expressed as mg gallic acid equivalents (GAE) g^-^¹ fresh weight.

#### 2.5.8 Total flavonoid content

The total flavonoids were estimated by the method of Zhishen et al. (1999). The extract was sequentially reacted with sodium nitrite, aluminum chloride, and sodium hydroxide. The absorbance was measured at 510 nm. Quercetin was used as the standard and the results were expressed in terms of mg quercetin equivalents (QE) per gram fresh weight.

#### 2.5.9 Chlorophyll and carotenoid content

Chlorophyll pigments were extracted using Arnon (1949). Fresh leaf tissue (100 mg) was homogenized in 80% acetone and then centrifuged. The absorbance of the supernatant was measured at 470, 645 and 663 nm. The contents of chlorophyll a, chlorophyll b, and total chlorophyll were determined by Arnon’s equations and presented as mg g^-^¹ fresh weight.

### 2.6 Preliminary compatibility assessment

Preliminary compatibility assessment of selenium nanoparticles (SeNPs) against *Pseudomonas fluorescens* and *Bacillus megaterium* was tested by the agar well diffusion method as reported by Cremonini et al. (2016). The bacterial cultures were uniformly spread on nutrient agar and the wells were filled with selenium nanoparticle suspension, and incubated at 28 ± 2 °C for 24–48 hours.

### 2.7 Statistical analysis

All data were analyzed using RStudio with R software (version 4.2.1). Treatment effects were evaluated by one-way analysis of variance (ANOVA), and mean comparisons were performed using Fisher’s Least Significant Difference (LSD) test at p ≤ 0.05.

## 3 Result and discussion

### 3.1 UV–Vis spectroscopy

**Figure 3:**
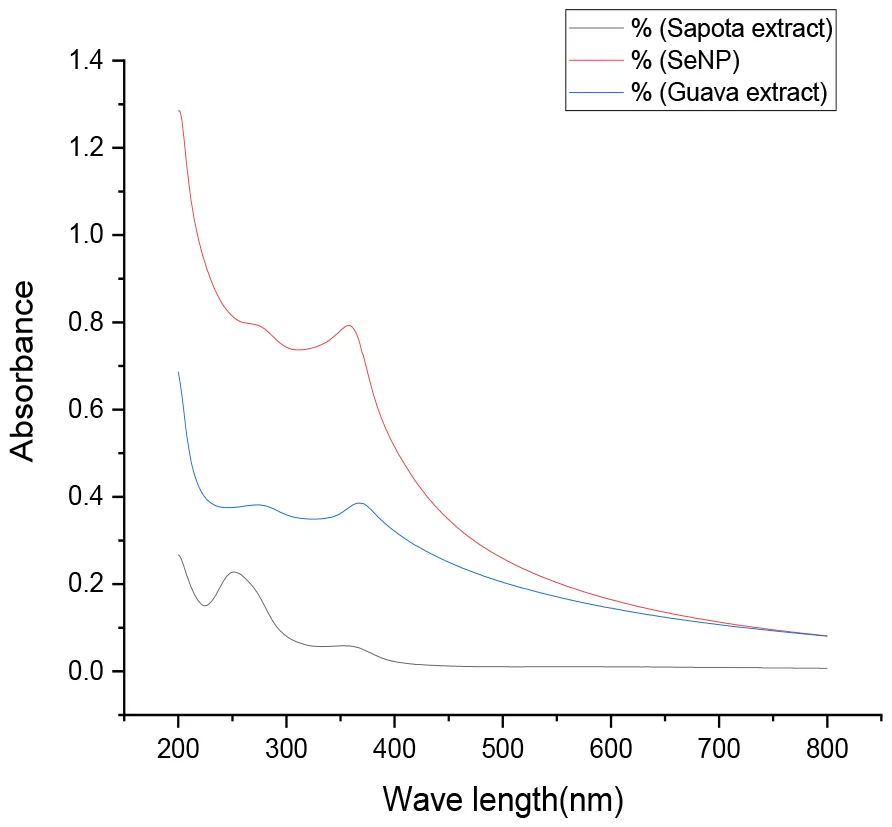
UV-Visible spectrum of SeNPs derived from guava leaf extract, sapota leaf extract and commercial SeNPs.

The UV-Visible absorption spectra (**Figure 3**) of the guava leaf extract, sapota leaf extract, and their respective selenium nanoparticles exhibited different absorption characteristics, which indicated effective formation of nanoparticles. Both plant extracts showed strong absorption between 200 and 300 nm, which is typical of 0-0, 0-H and N-H electronic transitions of phenolic compounds, flavonoids and other aromatic phytochemicals. When the nanoparticles were formed, the SeNP spectrum showed a larger and stronger absorption band between 250–400 nm, which is characteristic of elemental selenium nanoparticles. The same UV-vis absorption pattern has been observed with biogenic SeNPs produced by plant extracts such as Aloe vera, *Withania somnifera*, and *Psidium guajava*, indicating that the spectral variations exhibited are due to the reduction of selenium ions and the creation of nanoscale selenium (Wadhwani et al., 2016). The comparative analysis shows that the guava leaf-mediated SeNPs had a stronger absorbance intensity that might reflect the higher concentration of nanoparticles and the better dispersion stability. An increase in absorbance and spectral broadening of UV–Vis spectra are generally linked to an increased concentration of nanoparticles and enhanced interaction with phytochemicals bound to the surface. It is demonstrated in previous research that guava leaves have a greater level of polyphenols and antioxidants compared to sapota leaves which improves their reducing activity and stability during synthesis of nanoparticles (Eze et al., 2022). These UV-vis data are consistent with the XRD data where the amorphization is higher and FTIR data where the functional group interactions are stronger in guava-mediated SeNPs. The UV–Vis analysis also indicates that guava leaf extract is a better bioreductant and stabilizer, and produces SeNPs with better optical and structural characteristics that can be applied in agriculture.

### 3.2 FTIR

**Figure 4:**
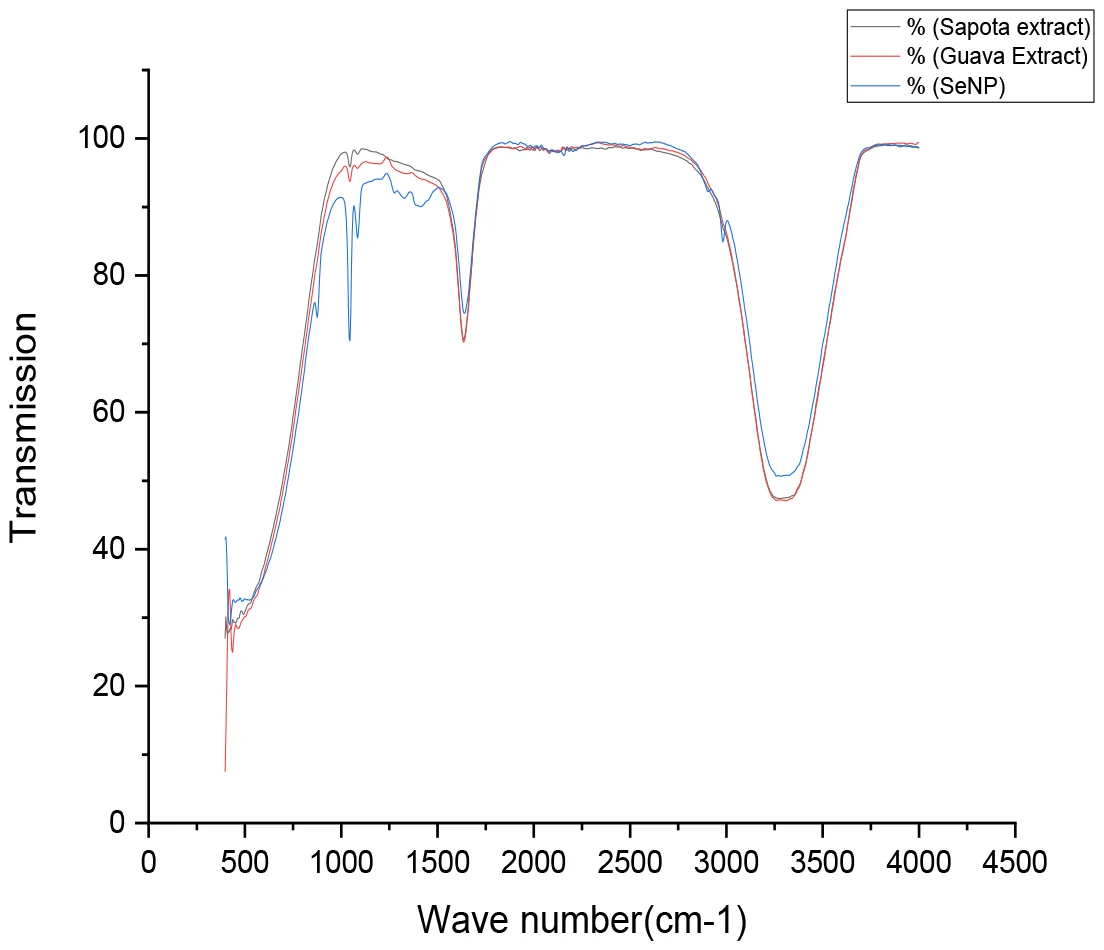
FTIR spectra of guava leaf extract, sapota leaf extract and SeNPs.

FTIR spectra of guava leaf extract, sapota leaf extract and their respective selenium nanoparticles (**Figure 4**) revealed the presence of a number of overlapping absorption bands which indicated the presence of plant-derived biomolecules used in green synthesis of SeNPs and their stabilization. The wide absorption at 3200–3400 cm^-^¹ in both extracts and SeNP samples is associated with O-H and N-H vibrations, which are typical of phenolics, alcohols, and proteins. Peaks around 1600–1650 cm^-^¹ are ascribed to C=O stretching (amide I) and C=C vibrations, which indicate the existence of proteins, flavonoids, and polyphenols. Also, bands at around 1380–1450 cm^-^¹ and 1000–1150 cm^-^¹ are C-N and C-O and carbohydrate functional groups. Plant-mediated SeNPs have been extensively reported as having similar FTIR signatures, which confirm that phytochemicals are reducing and capping agents in the formation of nanoparticles (Ramamurthy et al., 2019; Vahdati and Tohidi Moghadam, 2020). A comparative study indicates guava leaf-derived SeNPs had a slightly larger peak shifts and a greater intensity decrease than sapota-derived SeNPs, and thus, indicated better interaction between selenium and guava phytochemicals. These peak shifts and broadening upon formation of the nanoparticles suggests coordination between functional groups (–OH, -NH, -CO) and the surface of the selenium resulting in successful stabilization of the nanoparticles. It has been previously established that guava leaves contain elevated levels of phenolics and flavonoids compared to sapota leaves, and this increases their binding affinity and reducing efficacy (Pyrzynska and Sentkowska (2022); Abdulmumini et al., 2025). These results are consistent with the XRD and TGA results, as guava-mediated SeNPs exhibited higher amorphization and organic capping. The FTIR findings are a strong indication that guava leaf extract offers a better biochemical stabilization of SeNPs than sapota leaf extract, which substantiates its use in the agricultural field.

### 3.3 XRD

**Figure 5:**
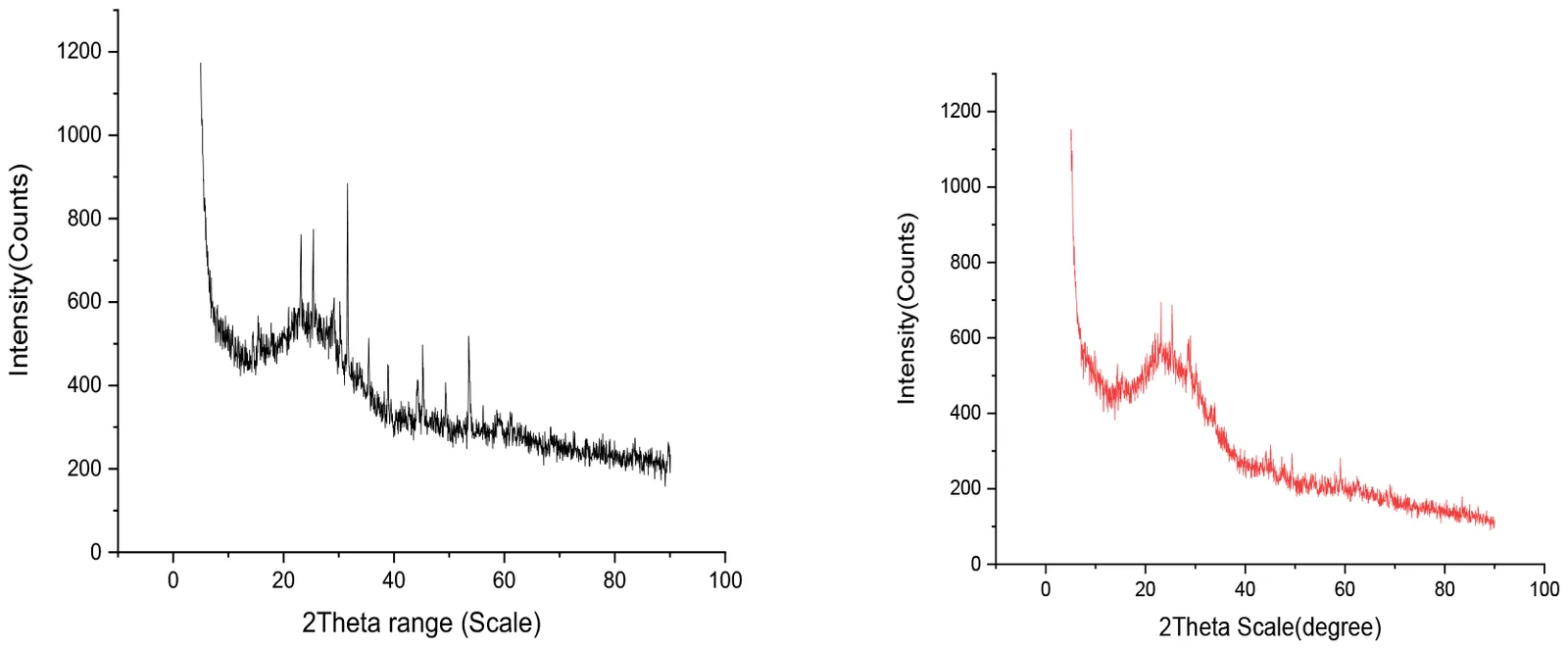
X- ray diffraction pattern of SeNPs from sapota leaf extract and guava leaf extract.

The XRD pattern shows the amorphous structure of the synthesized SeNPs (**Figure 5**) synthesized using both guava (*Psidium guajava*) and sapota *(Manilkara zapata*) leaf extracts, which is highly consistent with previous reports of plant-mediated synthesis of SeNPs. It has been shown that green-synthesized SeNPs do not commonly have sharp crystalline peaks because the nuclei of selenium and phytochemicals (polyphenols, flavonoids, tannins, and proteins) strongly interact, preventing crystal growth and causing structural disorder (Ramamurthy et al., 2019; Vahdati and Tohidi Moghadam, 2020). The diffraction hump in the 2θ ≈ 20–30° range is broad, indicating amorphous elemental selenium and is the same hump that has been repeatedly observed with SeNPs prepared using plant extracts such as Aloe vera, *Withania somnifera*, and *Crocus caspius* (Pyrzynska and Sentkowska (2022); Saravanan et al., 2025). It proves that the structural characteristics, which are revealed in the current work, are not accidental but a proven signature of biogenic selenium nanoparticles. In comparison, the guava leaf-based SeNPs had a stronger amorphous character as indicated by the wider and low-intensity diffraction patterns as compared to those obtained using sapota leaf extract. Phytochemical profiling studies have corroborated this finding with the report that guava leaves contain more phenolic acids, flavonoids, and antioxidant compounds compared than sapota leaves, thereby increasing their reducing and capping ability in the synthesis of nanoparticles (Abdulmumini et al., 2025). There is also an association between enhanced amorphization in SeNPs and enhanced biological activity, such as enhanced antioxidant activity, increased bioavailability, and enhanced interaction with biological systems, which are reported in previous studies (Wadhwani et al., 2016; Hosnedlova et al., 2018). Thus, the combination of current the XRD data and the current literature demonstrate that guava leaf extract offers better stabilization and structural control during the formation of SeNPs than sapota leaf extract. This improves the amorphous nature of the guava-mediated SeNPs and makes them better suited to agricultural applications, which supports the novelty and relevance of the present study.

### 3.4 FE-SEM with EDX

**Figure 6:**
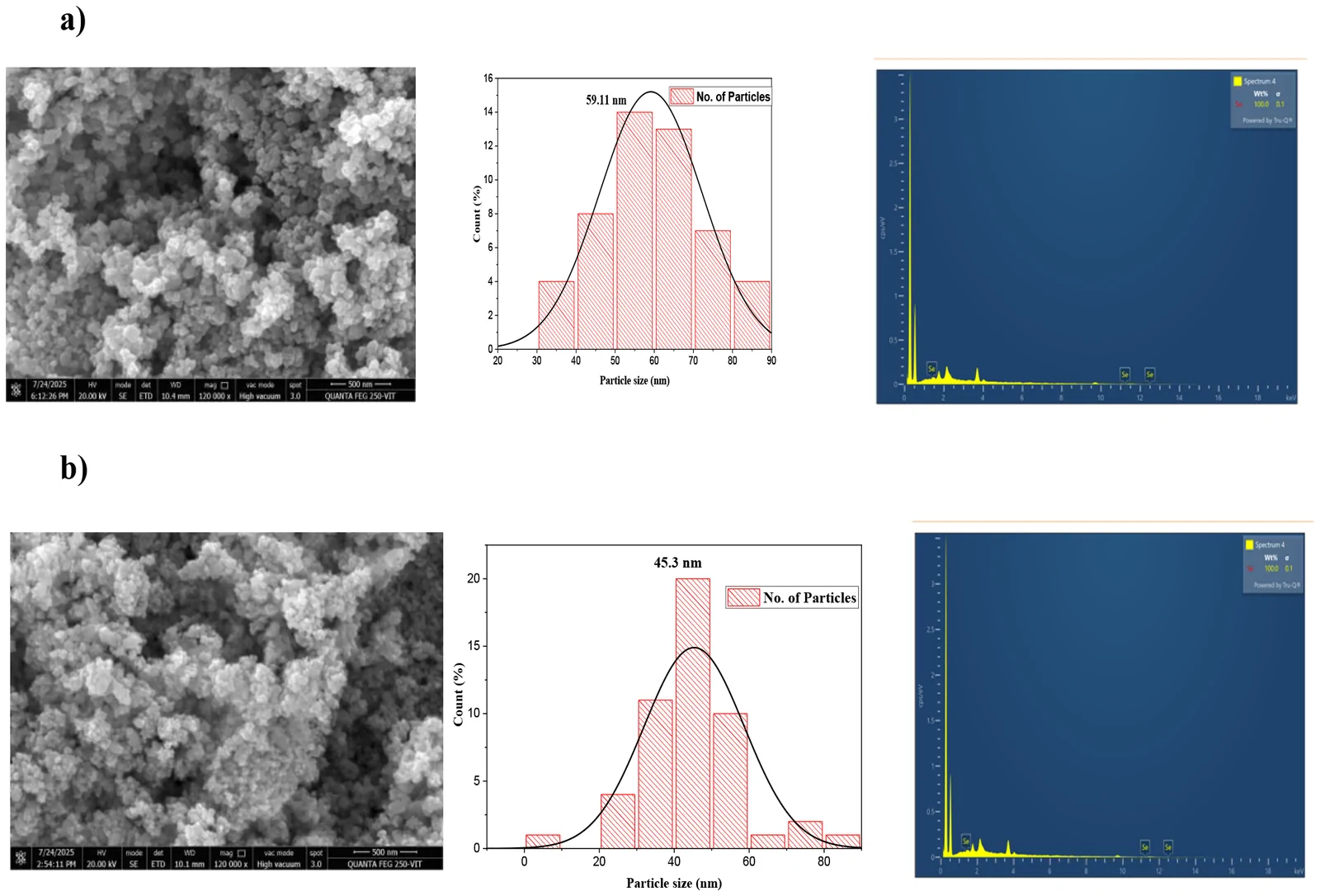
FE-SEM micrographs of green synthesized SeNPs derived from **(a)** Sapota leaf extract, **(b)** guava extract with their size distribution histogram and EDX analysis.

The analysis of the biosynthesized selenium nanoparticles by scanning electron microscopy (SEM) (**Figure 6**) showed a high concentration of almost spherical to quasi-spherical nanoparticles in the form of clustering and aggregation. The particles were evenly spread across the surface and had a typical morphology that is linked with plant-mediated synthesis pathways. The agglomeration observed may be explained by the large surface energy of nanoscale selenium particles and the presence of phytochemical capping agents in the leaf extract, which facilitates interparticle interactions. The presence of similar spherical and aggregated morphologies has been extensively reported in green-synthesized selenium nanoparticles with multiple plant extracts, which further substantiates the similarity of the current study with the existing data (Saravanan et al., 2025; Qin et al., 2025). The main size of the synthesized SeNPs was estimated to be about 45.59 nm based on the SEM micrograph obtained at 120,000 x magnification with a 500 nm scale bar. The estimation of the size was performed based on the comparison of the individual diameters of the particles with the scale bar and only the primary particles were counted using the scale bar and the secondary agglomerates were excluded. This nanoscale size is consistent with already reported biosynthesized selenium nanoparticles and adds to the amorphous nature of the XRD analysis. The relatively small size is beneficial in terms of biological and agricultural applications, with the smaller size increasing surface area, reactivity, and bioavailability. The elemental composition and purity of the synthesized nanoparticles were verified by energy-dispersive X-ray (EDAX) analysis, where several strong peaks of selenium were observed at typical energies of Se L and Se K transitions. The predomination of selenium signals and lack of major impurity peaks demonstrate that selenium ions are effectively reduced to elemental selenium nanoparticles. Small signals, if present, can be assigned to the carbon and oxygen provided by the organic capping agents or the sample holder. Combined with XRD, FTIR, UV-Vis, and TGA measurements, the SEM EDAX findings prove the green synthesis of stable and phytochemically capped selenium nanoparticles.

### 3.5 Zeta potential and particle size analysis

**Figure 7:**
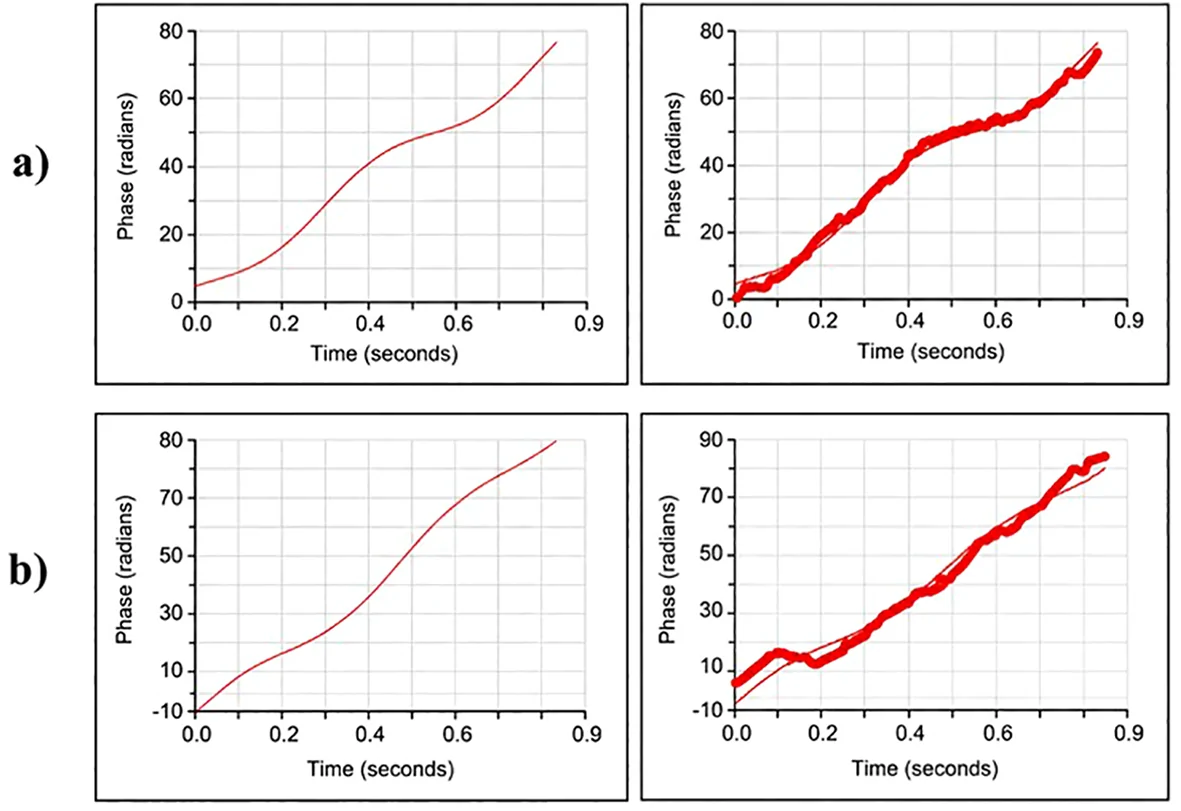
Zeta potential of green synthesized SeNPs derived from **(a)** guava leaf extract, **(b)** sapota leaf extract.

**Figure 8:**
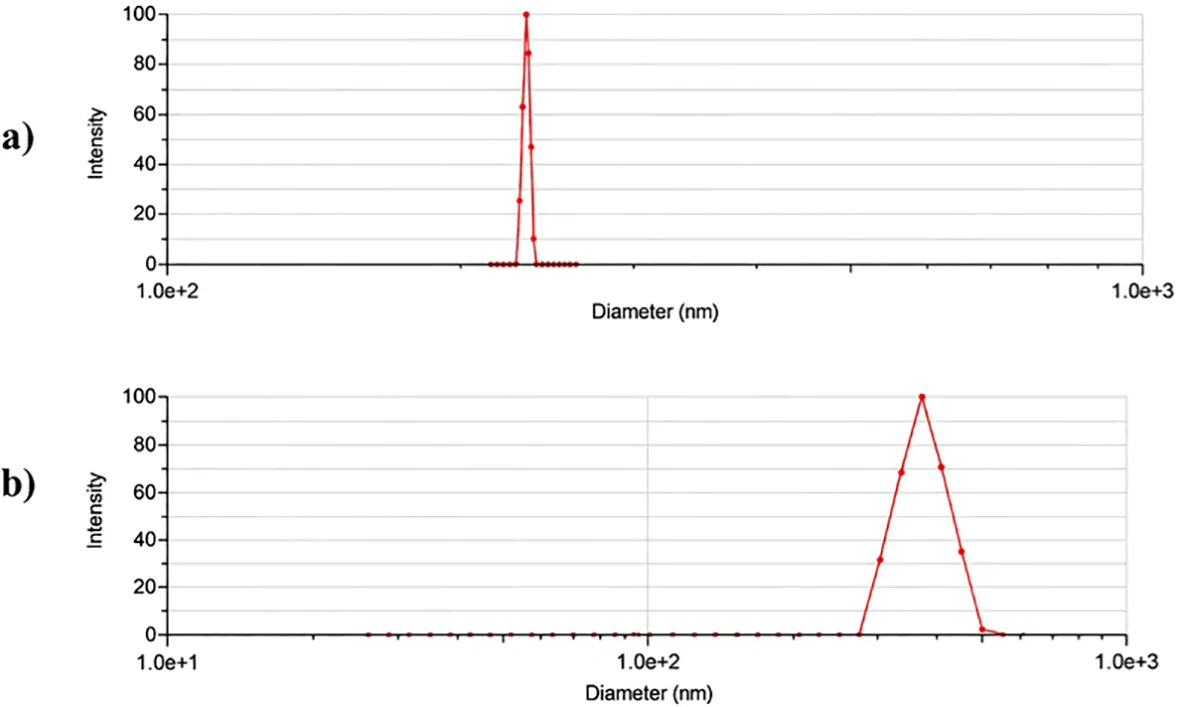
Particle size analysis of green synthesized SeNPs derived from **(a)** guava leaf extract, **(b)** sapota leaf extract.

Zeta potential analysis of selenium nanoparticles prepared with guava and sapota leaf extracts showed that the nanoparticles had a negatively charged surface, which implies that the nanoparticles were well stabilized in a colloidal suspension. The surface charge was observed to be due to the adsorption of biomolecules like phenolics, flavonoids, and proteins in the plant extracts that serve as natural capping and stabilizing reagents. The negative zeta potential values indicate that the particles are repelled on the basis of electrostatics, thus preventing aggregation and promoting the stability of colloidal particles (**Figure 7**). The same has been observed in other studies where phytochemical capped green-synthesized selenium nanoparticles were found to have negative surface charge and enhanced stability (Wang et al., 2020; Menon et al., 2018). Dynamic Light Scattering (DLS) is commonly employed to measure nanoparticle size distribution and hydrodynamic diameter, which indicates stability and aggregation behavior of particles in colloidal systems (Menon et al., 2018; Gunti et al., 2019). DLS analysis showed that selenium nanoparticles prepared with guava leaf extract had a smooth size distribution with a mean hydrodynamic diameter of about 120–150 nm, indicating a monodispersed and high stability. The DLS measurements of selenium nanoparticles prepared by sapota leaf extract revealed a larger size distribution with an average particle size of about 300–400 nm indicating polydispersity and potential aggregation (**Figure 8**).

### 3.6 TGA

**Figure 9:**
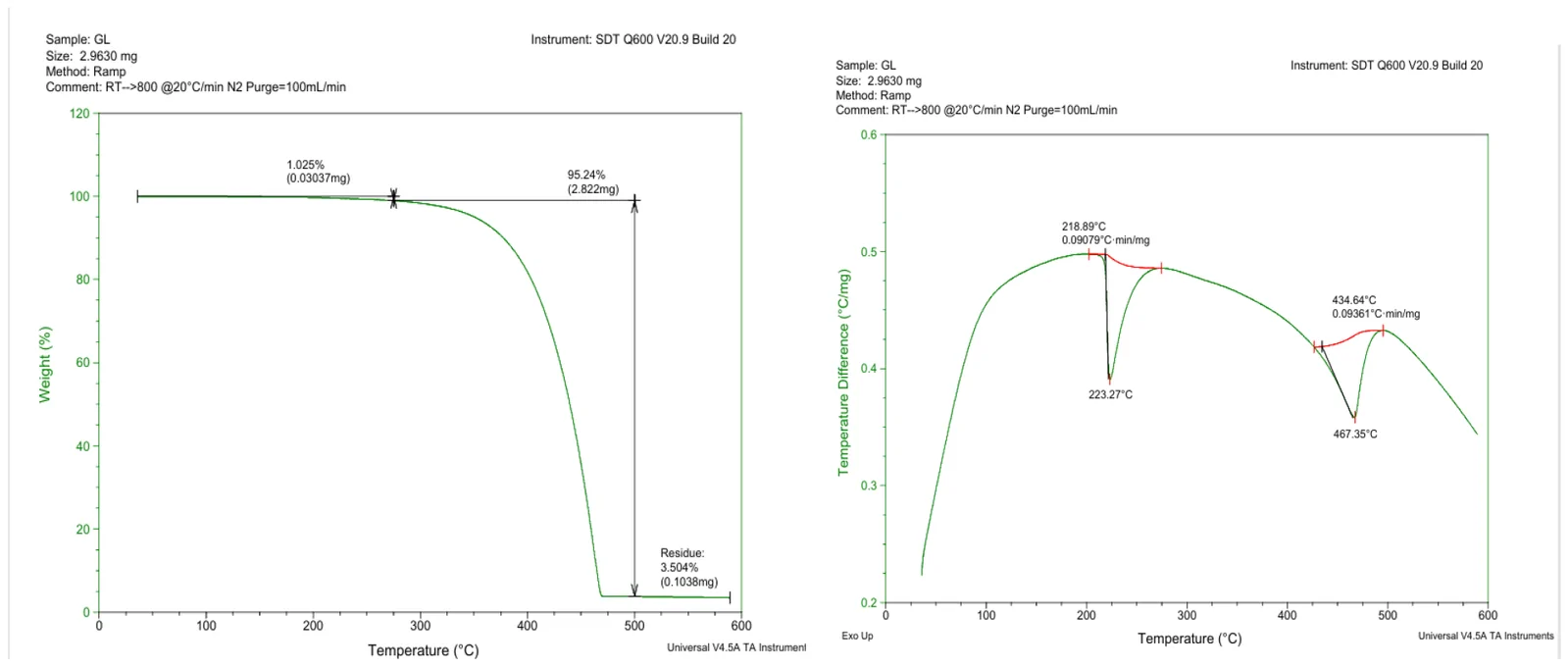
DTA and TGA result of SeNPs derived from guava extract.

Thermogravimetric analysis (**Figure 9**) of the biosynthesized selenium nanoparticles (SeNPs) revealed a characteristic multi-stage thermal degradation pattern that is consistent with previously reported plant-mediated SeNPs. The initial negligible weight loss (~1%) observed up to approximately 250 °C can be attributed to the removal of physically adsorbed moisture and weakly bound volatile phytochemicals. Similar low-temperature stability has been reported for SeNPs synthesized using various plant extracts, indicating effective surface stabilization by bioactive compounds present in leaf extracts (Qin et al., 2025). This thermal stability at lower temperatures confirms that the biomolecules involved in green synthesis form a protective organic layer around the selenium core, enhancing nanoparticle integrity. A major weight loss (~300–500 °C) was observed in the present study corresponds to the thermal decomposition of organic capping agents such as polyphenols, flavonoids, proteins, and carbohydrates derived from the leaf extract. Comparable decomposition regions have been reported for biosynthesized SeNPs using *Ficus platyphylla* leaf, bark extracts and other plant systems, where substantial mass loss in this temperature range was attributed to the breakdown of phytochemical coatings (Abdulmumini et al., 2025). The small residual mass (~3.5%) remaining beyond 500 °C represents thermally stable elemental selenium, confirming successful nanoparticle formation. The presence of multiple DTG peaks further supports a heterogeneous and multilayered organic capping structure, which is a typical feature of green-synthesized SeNPs and is considered beneficial for their stability and biological functionality.

### 3.7 Effects of SeNP seed priming on germination performance, morphophysiological traits in aggregatum onion under salinity stress

**Figure 10:**
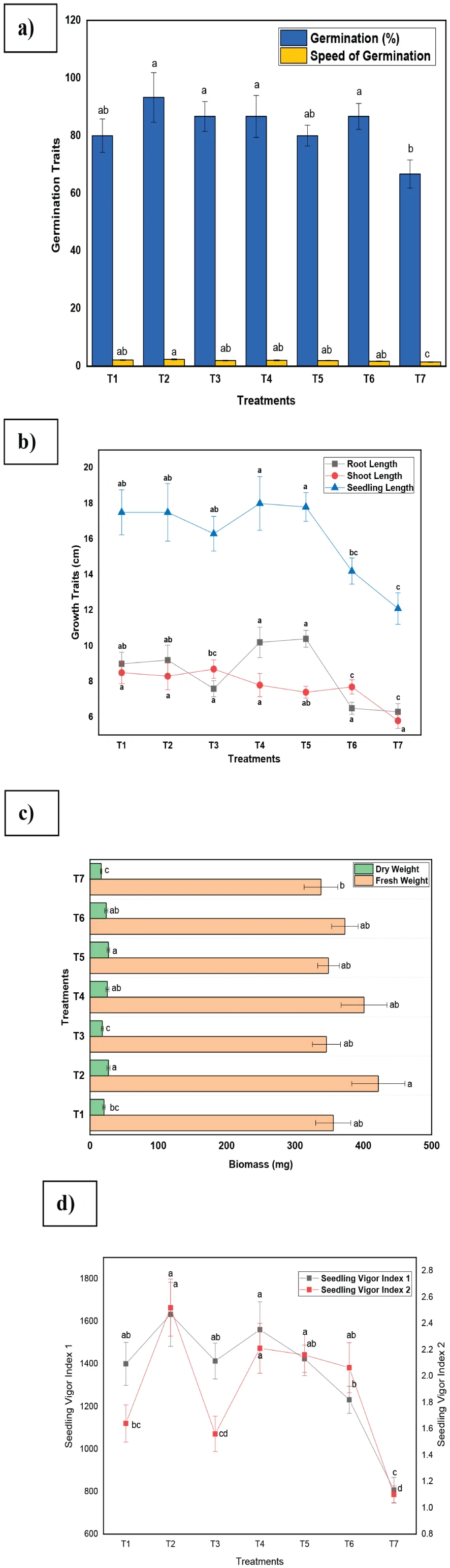
Effects of SeNPs on seed germination and seedling vigor **(a)**. germination percentage (GP%), speed of germination (SOG), **(b)** root length (cm), shoot length (cm), seedling length (cm); **(c)** fresh weight (mg), dry weight (mg); and **(d)** radar plot illustrating seed vigor index I and II under salinity stress. The lowercase letters represent the respective treatments T1-T7. T1: SeNPs @ 25 ppm; T2: SeNPs @ 50 ppm; T3: SeNPs @ 25 ppm + 25 mM NaCl; T4: SeNPs @ 50 ppm + 25 mM NaCl; T5: Control; T6: Hydropriming; and T7: 25 mM NaCl.

Seed germination is an important initial developmental phase for plant survival, and it is a critical stage in response to biotic and abiotic stresses, especially salinity (Singh et al., 2020). Salt stress affects various biochemical and physiological processes at all growth stages, including germination (El-Badri et al., 2021). Considering the adverse effects of salinity stress on rapeseed plants, researchers are developing salt-tolerance strategies using different approaches, including exogenous protectants to alleviate salt-induced oxidative stress in plants. In the present study, the significant application of the nanoparticles would be to reduce ionic toxicity and osmotic stress. Seedling development decreased with salt stress, likely because of ion toxicity and osmotic stress in the plants followed by nutrient imbalance and oxidative stress caused by excessive uptake of Na+ and Cl- which decreased the important mineral entry (Zhu, 2016). The aim of this study was to explore the impact of SeNPs on salt stress induced morpho-physiological processes in *Allium cepa* var. aggregatum during the early stage of seedling development. As the NPs are small in size, with a high surface area, they can easily penetrate into the cell, either by entering through the seed coat surface or through absorption with the texture parenchymatous, which results in an increase in the germination rate (Das et al., 2018). Plants have defense mechanisms including enzymatic and non-enzymatic antioxidants that protect cells from damage and maintain ROS levels within the cell by scavenging ROS (Shabala and Munns, 2017). The findings show that the selenium nanoparticles prepared with the help of guava leaf extract exhibited better stability and functionality. These SeNPs can be incorporated successfully into plant systems because of their homogenous nanometric size, favorable morphology, and presence of phytochemical capping agents, which together promote the controlled release of selenium, enhanced uptake efficiency, and promote plant growth under stress conditions (Wang et al., 2020; Qin et al., 2025; Islam et al., 2025). The germination test (**Figure 10**) actually indicated that the application of the selenium nanoparticles had a great enhancement effect on the germination and quality of the seedlings of the aggregatum onion compared with the control. SeNPs 50 ppm (T_2_) had the highest germination percentage (93.3%), and the lowest percentage of abnormal and deformed seedlings. This shows that optimally synthesized SeNPs were effective to promote seed metabolic activity and early seedling establishment. Conversely, T_7_ had the lowest rate of germination (66.7%), more number of abnormal seedlings, and the lowest seedling vigor validating the beneficial role of selenium supplementation during germination. The present findings agree with García-Locascio et al. (2024), who reported that SeNP seed priming significantly enhanced seed germination and seedling growth.

The growth parameters of the seedlings also proved the excellence of biogenic SeNPs over control and hydropriming. Root and shoot elongation, fresh weight, and dry biomass were significantly increased with treatments using SeNPs over control and hydropriming. It is observed that SeNPs at 50 ppm (T_2_) gave the highest root length (10.2 cm), GL-SeNP with 50 ppm (T_2_) gave the highest fresh (422 mg) and dry weight (27 mg), speed of germination (2.4), vigor index-1 (1632) and vigor index 2 (2.5) which indicate better nutrient assimilation and biomass accumulation, early germination, and greater vigor than other treatments including control. These advantages could be explained by the fact that SeNPs could control the activity of reactive oxygen species, induce the action of antioxidant enzymes, and promote cell division in early growth stages. Comparatively, treatments (T_5_ and T_6_) not only improved germination and seedling growth moderately but also revealed non-germinated and deformed seedlings, suggesting that there is a smaller safety margin of bulk forms of selenium. Consistent with the present study, Rangseekaew et al. (2026) demonstrated that biogenic SeNPs enhanced biomass accumulation and antioxidant defense under salt stress. The same was reported by Hernández-Hernández et al. (2019), who found that nano-selenium was less phytotoxic and more biologically efficient than selenate or selenite. Phytochemical capping of biogenic SeNPs by leaf extracts can also be attributed to the fact that phytochemicals increase the stability of the nanoparticles and their bioavailability (Singh et al., 2023). The current results are in line with the literature where low doses of SeNPs have been shown to promote germination and early seedling development of crops like wheat, soybean, sesame and rice, whilst elevated or excessive doses of selenium have led to growth retardation (Gunti et al., 2019; Li et al., 2024). Recent research by Mishra et al. (2025) showed that the seed treatments of nanoparticles enhance water uptake, enzyme activation, and antioxidant homeostasis during germination. On the same note, Gomathi et al. (2025) indicated that SeNPs that have been synthesized using green methods serve as nano-biostimulant agents in promoting photosynthetic efficiency, antioxidant defense, and biomass in Amaranthus microgreens. The findings show that selenium nanoparticles synthesized using guava leaf extracts, especially at 50 ppm, are superior to other treatments in enhancing the percentage of germination, seedling vigor, and biomass accumulation in aggregatum onion. This validates the possibility of biogenic SeNPs as a safe and effective nano-enabled approach to enhancing the quality of seeds and early crop establishment in the crop production.

### 3.8 Biochemical analysis

The application of NPs increased the enzymatic activity, as they are likely to increase the expression level of stress-responsive genes and ROS accumulation, thereby improving the development under stress (Das et al., 2018). Se induced the transcription of antioxidant defense-responsive genes that ultimately improved enzymatic activity in maize (Jiang et al., 2017). Additionally, SeNPs activate the antioxidant system and increase stress tolerance in plants (Hussein et al., 2019).

#### 3.8.1 SOD, POD and CAT

**Figure 11:**
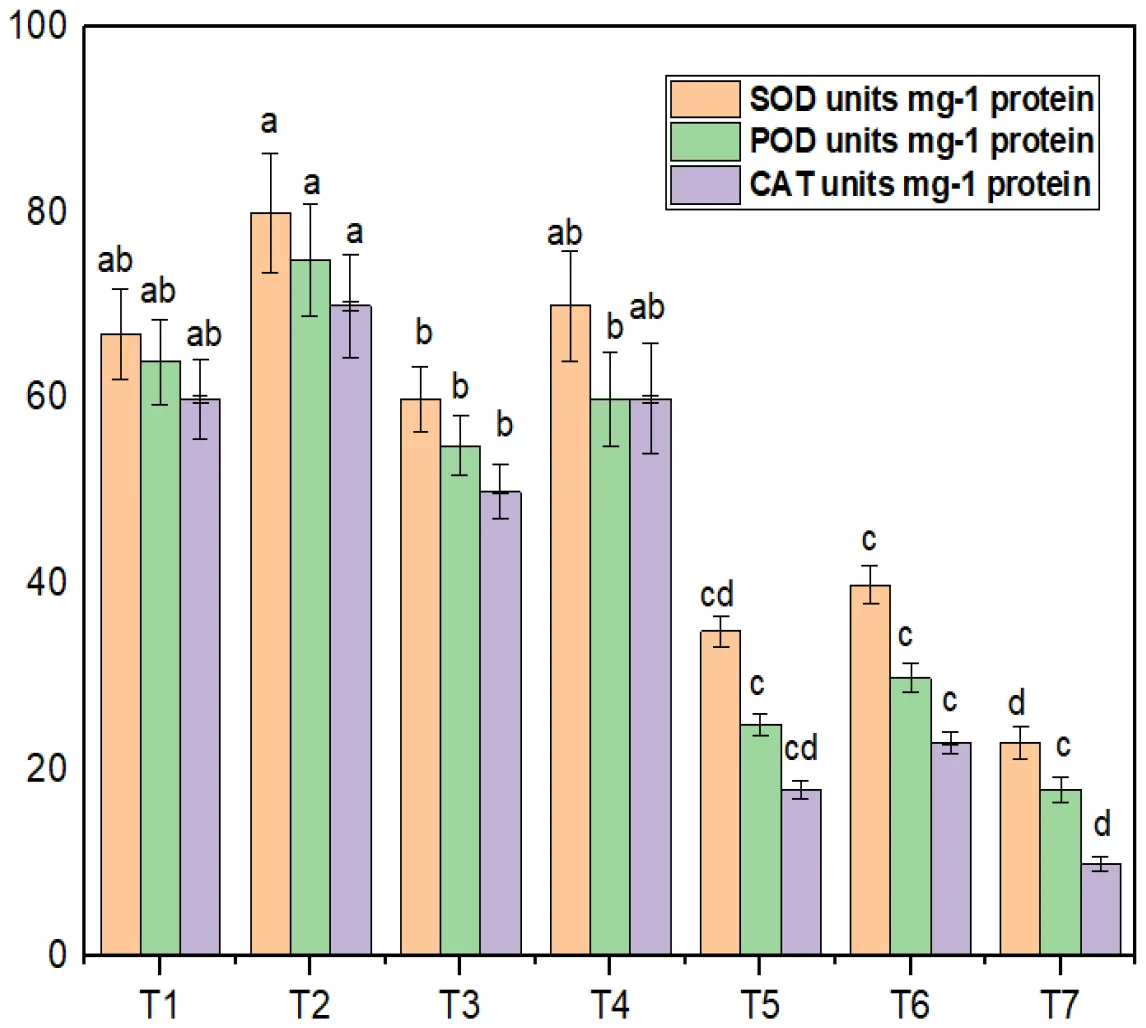
Effects of SeNPs on SOD, POD and CAT activity under salinity stress. The lowercase letters represent the respective treatments T1-T7. T1: SeNPs @ 25 ppm; T2: SeNPs @ 50 ppm; T3: SeNPs @ 25 ppm + 25 mM NaCl; T4: SeNPs @ 50 ppm + 25 mM NaCl; T5: Control; T6: Hydropriming; and T7: 25 mM NaCl.

The SOD activity was significantly increased in the SeNP treatments compared with the control and hydropriming treatments suggesting increased antioxidant activity and increased scavenging of superoxide radicals during stress conditions. The higher SOD activity was recorded in T_2_ (80 U mg^-^¹ protein) (**Figure 11**). Semida et al. (2021) have reported that the application of selenium enhanced the activity of SOD in onion under saline soil conditions. According to Hasanuzzaman et al. (2021), selenium boosts the detoxification of ROS by activating antioxidant enzymes. Elkelish et al. (2019) also showed that the antioxidant metabolism is enhanced by selenium, leading to better stress resistance. The findings are similar to that of Hussein et al. (2019) and Jiang et al. (2017) who found that SeNP application under abiotic stress resulted in an increase in SOD activity. The lowest SOD activity observed in T_7_ (23 U mg^-^¹ protein) could be due to high amounts of ROS generation and oxidative damage due to salinity stress, reducing the antioxidant defense systems. In onion, García et al. (2020) reported reduced activity of SOD under severe salinity stress, while Astaneh et al. (2019) reported a similar finding in garlic. Sanwal et al. (2022) have reported that under severe salinity stress, the activity of SOD decreased in Allium species, and several vegetable crops also reported reduced activity of SOD under severe salinity stress (Hasanuzzaman et al., 2021; Elkelish et al., 2019).

The POD activity was significantly higher in the SeNP treatments than in the control and hydropriming treatments, which is evidence of better antioxidant defense and better detoxification of the hydrogen peroxide accumulated under salinity stress. The higher POD activity was recorded in T_2_ (75 U mg^-^¹ protein) (**Figure 11**). Peroxidase is one of the most important antioxidants that belong to the enzymatic antioxidant system, which helps to neutralize the effect of the so-called free radicals (reactive oxygen species), which may cause oxidative damage to the cellular structure. Selenium application has been reported to induce a similar increase in POD activity in onion under saline conditions by Semida et al. (2021). Hasanuzzaman et al. (2021) proposed that the positive effects of selenium on ROS detoxification may be due to its induction of antioxidant enzymes such as POD, which can increase abiotic stress tolerance in plants. Similarly, Xing et al. (2024) demonstrated that SeNP seed priming improved salinity tolerance by enhancing antioxidant defense and maintaining redox homeostasis. Elkelish et al. (2019) also showed that selenium-mediated antioxidant metabolism is associated with enhanced protection of cells and resistance to stress. Similarly, Hussein et al. (2019) noted that the application of selenium nanoparticles under salt stress led to higher POD activity, and Jiang et al. (2017) found that under salinity stress, SeNP application was efficient in enhancing the antioxidant defense system by increasing the activity of POD in maize plants. However, the salinity-induced POD activity recorded in T_2_ (18 U mg^-^¹ protein) can be attributed to increased oxidative stress and cellular damage, which affected the functioning of antioxidant enzymes. García et al. (2020) reported salt stress-induced reduction in POD activity in onion, Astaneh et al. (2019) observed the same in garlic and Sanwal et al. (2022) reported in Allium species under salinity stress. The results indicate that SeNP promotes the POD-mediated ROS scavenging that results in an increase in salinity stress tolerance in aggregatum onion seedlings.

The increased CAT activity found in SeNP treatments compared with the control and hydropriming treatments reflects its ability to break down hydrogen peroxide, as well as its ability to maintain cellular redox balance. Enhancement of CAT activity has been widely reported as being associated with stress tolerance. The higher POD activity was recorded in T_2_ (70 U mg^-^¹ protein) (**Figure 11**). Liu et al. (2022) reported that selenium promotes antioxidant enzymes that detoxify ROS. Ishtiaq et al. (2023) reported that nano-selenium application led to an increase in CAT activity in stressed plants. In the study by Mishra et al. (2025), the CAT activity was significantly increased in plants under salinity stress when treated with SeNP. The effect of nano-selenium on enhancing CAT activity under drought conditions was also shown by Zeeshan et al. (2024). Arivalagan and Das (2026) also highlighted the importance of SeNPs in enhancing the antioxidant metabolism and oxidative stress protection. The decrease in CAT activity recorded in T_7_ (10 U mg^-^¹ protein) (**Figure 11**) indicates extreme stress caused by increased ROS levels due to salinity exposure and its subsequent detoxification. The present findings agree with Roy et al. (2025b), who reported that ZnO nanoparticles alleviated salinity-induced oxidative stress and improved plant performance under saline conditions, supporting the role of nanoparticles in enhancing stress tolerance.

#### 3.8.2 Total phenol, flavonoids and proline content

**Figure 12:**
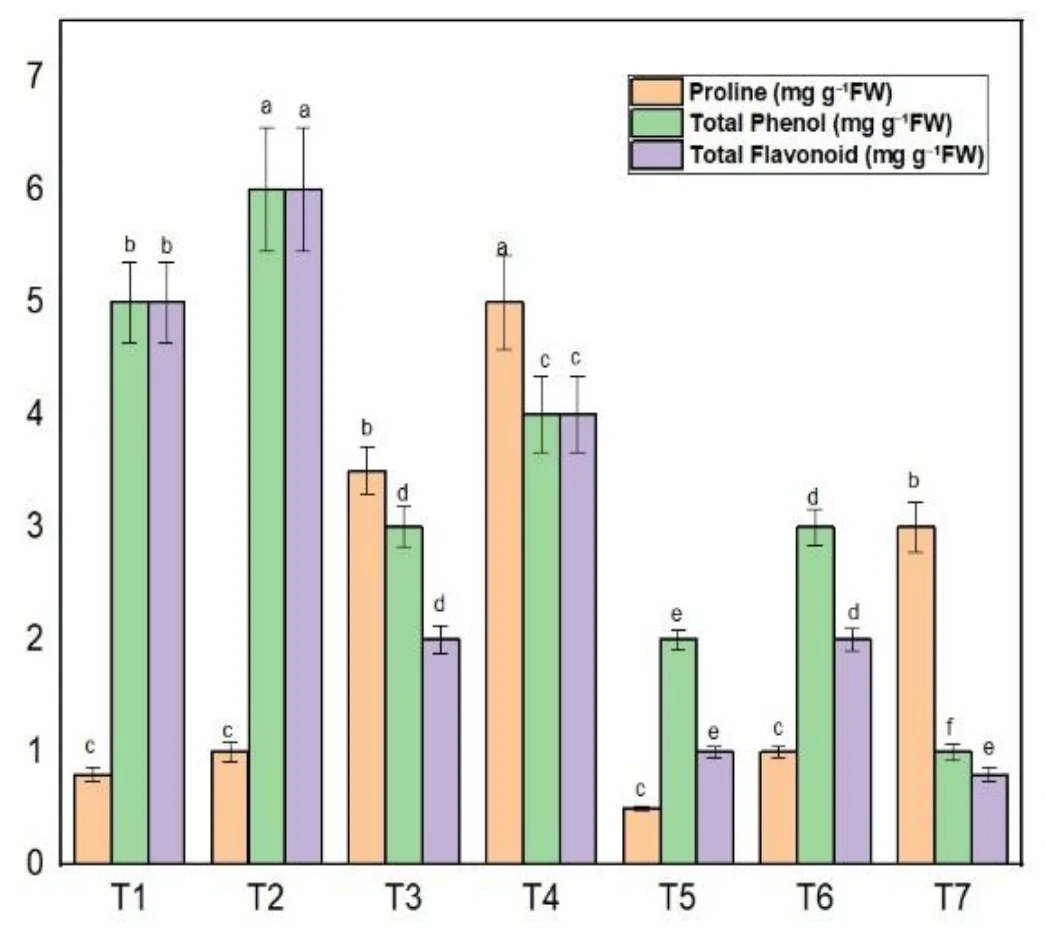
Effects of SeNPs on total phenol, flavonoids and proline content under salinity stress. The lowercase letters represent the respective treatments T1-T7. T1: SeNPs @ 25 ppm; T2: SeNPs @ 50 ppm; T3: SeNPs @ 25 ppm + 25 mM NaCl; T4: SeNPs @ 50 ppm + 25 mM NaCl; T5: Control; T6: Hydropriming; and T7: 25 mM NaCl.

The total phenol content was determined, and increased phenolics was found in SeNP treated seedlings as compared to the control and hydropriming treatments (**Figure 12**). The maximum total phenol content was observed in treatment T_2_ (6 mg g-1) suggests a higher biosynthesis of phenolic compounds, which are important non-enzymatic antioxidants that scavenge the reactive oxygen species (ROS) and protecting the cell components from oxidative stress due to salinity stress. Phenolic compounds play important roles in membrane stability, stress adaptation and maintaining cellular redox homeostasis. Supporting the present findings, Ameen et al. (2024) showed that selenium application enhanced total phenolic content, thereby improving antioxidant activity and stress tolerance in sunflower. The same conclusions have been drawn by several studies. Hawrylak-Nowak et al. (2015) found that cucumber plants exposed to selenium showed an increase in phenolic compounds and antioxidant activity. Similarly, Skrypnik et al. (2019) reported that the application of Se significantly improved total phenolic content and antioxidant capacity of basil plants. Osman et al. (2026) similarly reported that green-synthesized SeNPs improved growth, antioxidant activity, and salinity tolerance in seedlings. In addition, El-Badri et al. (2021) found that Selenium nanoparticles positively affected antioxidant metabolism and stress tolerance by regulating the biosynthesis of secondary metabolites in a salinity stress environment. The lowest phenol content was observed in T_7_ (1 mg g-1). However, this could be related to the presence of high levels of oxidative stress that may lead to reduced biosynthesis of phenols and depletion of antioxidant metabolites. The present study indicated that SeNP priming enhanced the total phenol content which was correlated with the improvement in non-enzymatic antioxidant defense mechanisms and thus increased the salinity stress tolerance of the seedlings.

Flavonoids are potent free radical scavengers and are also crucial in the defense of membranes and biomolecules from oxidative damage. The higher concentration of flavonoids in treatment T_2_ (6 mg g^-^¹) suggests greater production of non-enzymatic antioxidants which are important in ROS detoxification and protection of cells under salinity stress. Similarly, Zhao et al. (2024) reported that selenium treatment promoted flavonoid accumulation and enhanced antioxidant capacity in mungbean sprouts by regulating flavonoid biosynthesis, thereby improving stress tolerance. Wang et al. (2020) reported that selenium treatment promoted the accumulation of flavonoids and antioxidant metabolism in alfalfa via the regulation of phenylpropanoid pathways. According to Skrypnik et al. (2019), selenium supplementation increased flavonoid content and strengthened the antioxidant defense system in basil plants. Hasanuzzaman et al. (2021) found that SeNPs promoted the production of antioxidant metabolites, such as flavonoids, under drought stress. Liu et al. (2024) showed that applying nanoparticles of selenium enhanced the production of antioxidant compounds and stress resistance in crop plants. In a similar study, under environmental stress conditions, El-Batal et al. (2023) observed higher levels of flavonoids in carrots exposed to selenium nanoparticles. Thus, the enhanced level of flavonoids observed in the present study indicated that the priming of SeNP resulted in induction of non-enzymatic antioxidant defense mechanism, making them more tolerant against salinity-induced oxidative stress. However, the lower level of flavonoids in T_7_ (0.8 mg g^-^¹) may be related to the oxidative stress and the inhibition of secondary metabolite biosynthesis under salinity stress.

Selenium nanoparticle priming under salinity stress significantly affected the content of proline (**Figure 12**). The highest proline content was obtained in T_2_ (5 mg g-1) and the lowest content was obtained in T_5_ (0.5 mg g-1). Proline is a major compatible osmolyte that accumulates in plants under abiotic stress conditions and is important in osmotic adjustment, protein and membrane stabilization, protection of cell structure and scavenging of reactive oxygen species (ROS). Results of the enhanced accumulation of proline in SeNP-treated seedlings suggest better osmotic regulation and stress adaptation in saline conditions. This has been reported in a number of studies. Hasanuzzaman et al. (2020) indicated that application of selenium led to increased proline content and osmotic balance under abiotic stress conditions in plants. Similarly, El-Badri et al. (2021) found that SeNP priming resulted in the highest proline content and greatest salinity tolerance in rapeseed seedlings, attributed to enhancing antioxidant defense and osmotic adjustment. In the same way, Ikram et al. (2020) showed that selenium nanoparticles synthesized using plants effectively enhanced the proline content of wheat under drought stress, which in turn helped to alleviate drought-induced damage to cell. Furthermore, Hawrylak-Nowak (2008) found that there was an increase in the synthesis of proline and an enhanced tolerance to environmental stress conditions in plants supplemented with selenium. It is possible that the lower proline content of T_7_ (3 mg g−1) was due to the presence of severe metabolic impairment under salinity stress and hence less osmolyte accumulation and stress tolerance. Therefore, the higher proline level in the present study indicates that the primed seedlings were able to withstand osmotic stress and oxidative stress caused by salinity stress due to an effective enhancement of the osmoprotective mechanisms.

#### 3.8.3 MDA and H_2_O_2_ activity

**Figure 13:**
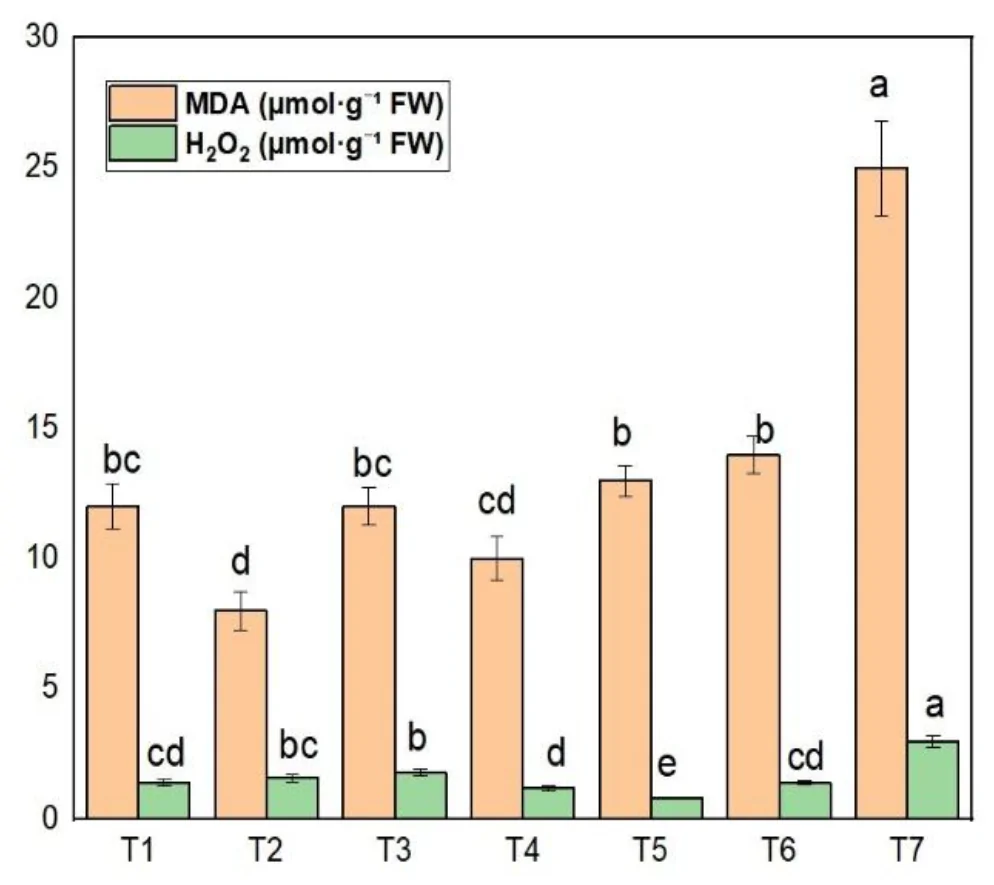
Effects of SeNPs on MDA and H_2_O_2_ activity under salinity stress. The lowercase letters represent the respective treatments T1-T7. T1: SeNPs @ 25 ppm; T2: SeNPs @ 50 ppm; T3: SeNPs @ 25 ppm + 25 mM NaCl; T4: SeNPs @ 50 ppm + 25 mM NaCl; T5: Control; T6: Hydropriming; and T7: 25 mM NaCl.

A lower MDA content in T_2_ (8 μmol g^-^¹FW) indicates less lipid peroxidation and increased membrane stability after SeNP application. Increased antioxidant protection has been commonly correlated with the selenium-mediated decrease in MDA accumulation. The SeNP treated plants showed lower MDA content than the control, Hydropriming and the salinity stress treatment (**Figure 13**). Consistent with the present findings, Desouky et al. (2024) reported that selenium nanoparticles significantly reduced MDA accumulation by protecting membrane integrity and alleviating oxidative damage under salinity stress in soybean. Ishtiaq et al. (2023) reported a decrease in oxidative membrane damage after application of nano-selenium. In salinity stress, onion plants exposed to selenium showed reduced accumulation of MDA, as reported by Semida et al. (2021). Mishra et al. (2025) found that the content of MDA decreased significantly in the plants treated with SeNP under salinity stress. The study by Zeeshan et al. (2024) demonstrated a significant reduction in the accumulation of MDA in drought-stressed plants. Liu et al. (2022) reported that selenium is protective against maintaining the integrity of membranes under environmental stress. Additionally, Arivalagan and Das (2026) reported that SeNPs can protect against oxidative injury by regulating antioxidant status. The highest MDA content was recorded in T_7_ (25 μmol g^-^¹FW) which shows that salinity stress caused severe damage to the membrane due to excessive ROS production. Increased MDA accumulation has been reported under salt stress in onion (García et al., 2020), garlic (Astaneh et al., 2019) and some horticultural crops under salt stress with an oxidative response (Hasanuzzaman et al., 2021; Elkelish et al., 2019).

The priming of selenium nanoparticles under salinity stress had a significant effect on hydrogen peroxide (H_2_O_2_) content (**Figure 13**). The highest H_2_O_2_ content was obtained in treatment T_7_ (3 μmol g^-^¹FW) while the lowest H_2_O_2_ content was obtained in treatment T_5_ (0.8 μmol g^-^¹FW). The production of hydrogen peroxide is one of the major reactive oxygen species (ROS) that occurs in plants under salinity stress and its overproduction may result in oxidative damage to the membranes, proteins, nucleic acids and the photosynthetic system of plants. Thus, the concentration of H_2_O_2_ is considered a good marker for the intensity of oxidative stress in plants. A considerable decrease in the H_2_O_2_ level was observed in T_5_, thus indicating that SeNP priming improved the ROS scavenging system and reduced the oxidative damage induced by salinity stress. This is consistent with previous studies. El-Badri et al. (2021) showed that the accumulation of H_2_O_2_ in salt-stressed rapeseed seedlings was drastically decreased due to the efficacy of selenium nanoparticles in improving the antioxidant defense system and detoxifying ROS. Similarly, bio-fabricated selenium nanoparticles reduced the level of H_2_O_2_ in wheat plants under drought stress which reduced the oxidative stress and enhanced the resistance of wheat plants to drought stress (Ikram et al., 2020). Hasanuzzaman et al. (2021) also found that the selenium increased the plants’ antioxidant defense system and decreased H_2_O_2_ accumulation under abiotic stresses. Likewise, Habibi (2013) reported that H_2_O_2_ contents in barley under drought stress were reduced when selenium was applied, as it improved antioxidant metabolism and decreased the membrane injury. The maximum H_2_O_2_ level in T_7_ (3 μmol g^-^¹FW) might be explained by the high level of oxidative stress and the lack of antioxidant protection, which causes ROS to accumulate and cause damage to the cells. Therefore, the SeNP priming was found to be effective in the amelioration of salinity-induced oxidative stress in the present study by its antioxidant activity and maintenance of cellular redox homeostasis.

#### 3.8.4 Chlorophyll and carotenoid content

**Figure 14:**
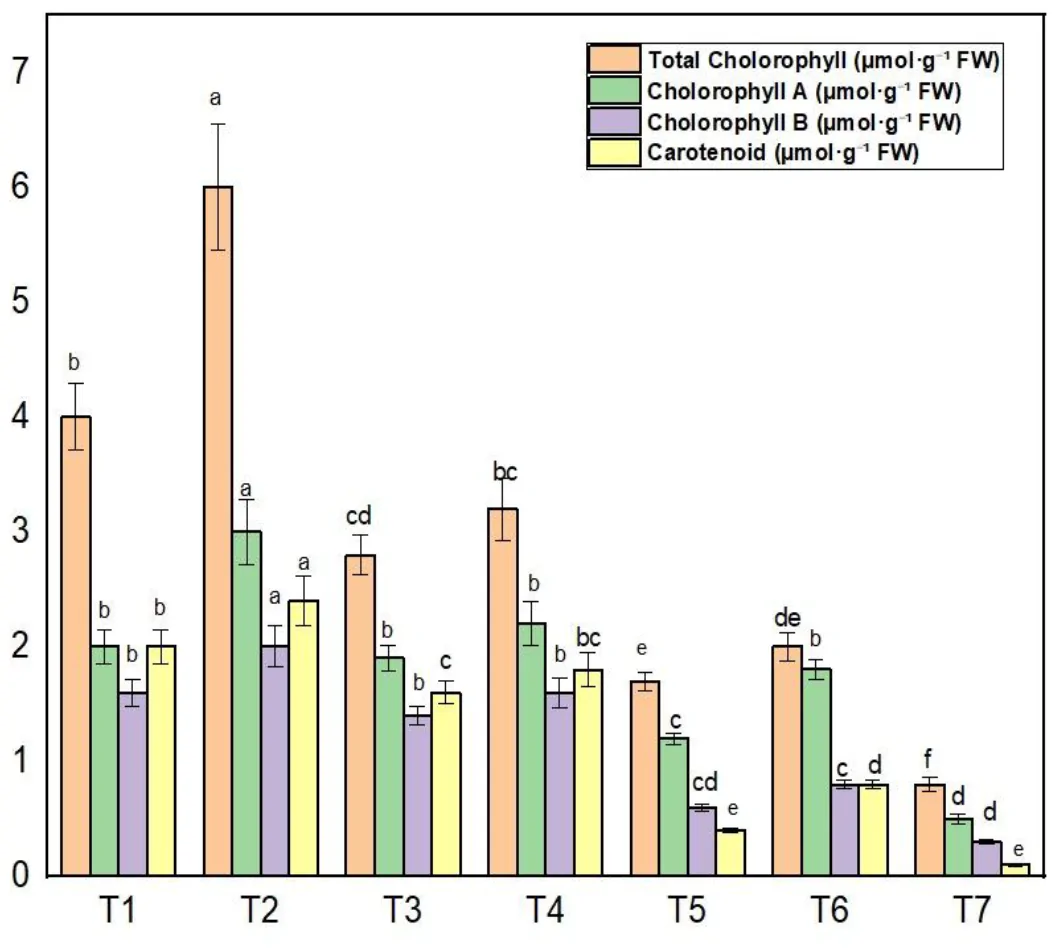
Effects of SeNPs on chlorophyll and carotenoid content under salinity stress. The lowercase letters represent the respective treatments T1 -T7. T1: SeNPs @ 25 ppm; T2: SeNPs @ 50 ppm; T3: SeNPs @ 25 ppm + 25 mM NaCl; T4: SeNPs @ 50 ppm + 25 mM NaCl; T5: Control; T6: Hydropriming; and T7: 25 mM NaCl.

The chlorophyll a, chlorophyll b, total chlorophyll and carotenoid content measured in SeNP treatments were found to be significantly higher when compared to control and hydropriming treatments, thus showing that the selenium nanoparticles could effectively protect the photosynthetic apparatus from the salinity-induced oxidative damage. The highest chlorophyll a (3 mg g^-^¹), chlorophyll b (2 mg g^-^¹), total chlorophyll (6 mg g^-^¹) and carotenoid content (2.4 mg g^-^¹) were measured in T_2_ (**Figure 14**). Stress-induced chlorophyll maintenance is crucial for maintaining plant growth and photosynthetic efficiency. Under drought stress, Hasanuzzaman et al. (2021) reported that the application of selenium nanoparticles significantly boosted the retention of chlorophyll and photosynthetic efficiency in wheat. Under water deficit conditions, Zeeshan et al. (2024) showed that SeNP application led to an increase in chlorophyll content and enhanced photosynthetic efficiency in soybean. Freire et al. (2024) showed that selenium nanoparticles increased the content of photosynthetic pigment and the physiological performance of rice seedlings Borbély et al., 2021. Under abiotic stress, Liu et al. (2024) reported that selenium nanoparticles treatment enhanced the stability of chlorophyll and increased photosynthetic activity (Borbely et al., 2021). Similarly, El-Batal et al. (2023) found that selenium nanoparticles protected photosynthetic pigments and enhanced physiological performance in carrot plants under environmental stress. Thus, the results of the present study show a higher chlorophyll content, which means that the damage caused by salinity stress to chloroplast structures was alleviated by SeNP and the photosynthetic activity was maintained. These findings are consistent with reports of Sierra et al. (2025) that SeNPs enhance seedling growth, chlorophyll content, and stress tolerance in crop plants. The lowest chlorophyll a (0.5 mg g^-^¹), chlorophyll b (0.3 mg g^-^¹), total chlorophyll (0.8mg g^-^¹) and carotenoid content (0.1 mg g^-^¹) were recorded in T_7_, however, could be due to salinity induced chlorophyll degradation and damage to the photosynthetic apparatus. Reduced chlorophyll levels have been reported in onion (García et al., 2020), garlic (Astaneh et al., 2019) and several vegetable crops exposed to salinity stress (Hasanuzzaman et al., 2021).

### 3.9 Preliminary compatibility assessment

The test showed that the guava leaf extract-based selenium nanoparticles (SeNPs) were not toxic to the beneficial rhizobacterial strains *Pseudomonas fluorescens and Bacillus megaterium.* The presence of active bacterial growth around the wells containing selenium nanoparticles on nutrient agar plates was observed with no inhibition zones. *Pseudomonas fluorescens* showed uniform and dense bacterial growth around the treated area, suggesting that the synthesized SeNPs did not adversely affect the viability of bacteria. Similarly, *Bacillus megaterium* was observed to have a healthy colony morphology and continuous growth in the vicinity of the application site of the nanoparticles, further signifying the non-toxicity of the nanoparticles toward the tested beneficial microorganisms. The lack of inhibitory zones suggests that the green synthesized selenium nanoparticles were non-toxic to the growth of beneficial soil bacteria. This could be due to the phytochemical constituents of guava leaf extract that play a role in the reduction and stabilization during the synthesis of nanoparticles, which results in reduced toxicity of the nanoparticles. Consistent findings were reported by Cremonini et al. (2016), who claimed that selenium nanoparticles produced by biological methods were less toxic and more biocompatible than nanoparticles produced chemically. Similarly, the study conducted by Menon et al. (2018) revealed that the plant-mediated selenium nanoparticles demonstrated high environmental safety and reduced negative impacts on microbial beneficial systems (Menon et al., 2018). In another study, Kokila et al. showed that the physicochemical properties of green synthesized selenium nanoparticles were found to be stable with minimal toxicity to biological systems due to the presence of natural bioactive compounds associated with plant extracts (Kokila et al., 2017). Thus, the present study demonstrates that the guava leaf extract mediated selenium nanoparticles are safe for use with beneficial plant growth promoting rhizobacteria and can be safely employed in the agricultural applications including biofertilizer formulation, plant growth promotion, and sustainable crop production systems, which involve use of nanoparticles.

## 4 Conclusion

In this work, we showed a scientific approach at a lower cost by applying nanoparticles, trying to avoid the use of chemicals that might be toxic to the environment, and also using an increasingly advanced seed germination under salinity stress, which would give greater promise for the use of nanoparticles applications on a larger scale. Detailed physicochemical characterization ensured the presence of SeNPs. The seed germination test provided a clearer picture of the high performance of the biogenic SeNP priming in comparison with the hydropriming and the control. Overall, our findings indicated a promising effect of nanopriming on increasing seed germination and antioxidant enzyme activity in *Allium cepa* var. aggregatum under salt stress conditions. These findings demonstrate the potential of guava-mediated SeNP seed priming under laboratory conditions, while emphasizing that greenhouse, pot, and field studies are required to validate its practical applicability before recommending agricultural use. Further research is needed to improve the efficiency of nanoparticles, including research on the priming time, optimal dose and the correlation between the properties of the nanomaterials and different physiological and developmental processes. The paper emphasizes the prospects of biogenic SeNPs to enhance seed germination and early seedling development in vegetable crops. These findings lead to future studies aiming at validating the field-scale, long-term performance of plants, and the use of SeNPs to alleviate stress and nutritional biofortification across different agro-ecological conditions.

## Data Availability

The raw data supporting the conclusions of this article will be made available by the authors, without undue reservation.
